# Deep learning–radiomics integrated noninvasive detection of epidermal growth factor receptor mutations in non-small cell lung cancer patients

**DOI:** 10.1038/s41598-024-51630-6

**Published:** 2024-01-09

**Authors:** Seonhwa Kim, June Hyuck Lim, Chul-Ho Kim, Jin Roh, Seulgi You, Jeong-Seok Choi, Jun Hyeok Lim, Lucia Kim, Jae Won Chang, Dongil Park, Myung-won Lee, Sup Kim, Jaesung Heo

**Affiliations:** 1 Department of Radiation Oncology, Ajou University School of Medicine, Suwon, Republic of Korea; 2 Department of Otolaryngology, Ajou University School of Medicine, Suwon, Republic of Korea; 3 Department of Pathology, Ajou University School of Medicine, Suwon, Republic of Korea; 4 Department of Radiology, Ajou University School of Medicine, Suwon, Republic of Korea; 5 Department of Otorhinolaryngology-Head and Neck Surgery, Inha University College of Medicine, Incheon, Republic of Korea; 6 Division of Pulmonology, Department of Internal Medicine, Inha University College of Medicine, Incheon, Republic of Korea; 7 Department of Pathology, Inha University College of Medicine, Incheon, Republic of Korea; 8 Department of Otolaryngology-Head and Neck Surgery, Chungnam National University Hospital, Daejeon, Republic of Korea; 9 Division of Pulmonary, Allergy and Critical Care Medicine, Critical Care Medicine, Department of Internal Medicine, Chungnam National University Hospital, Daejeon, Republic of Korea; 10 Division of Hematology and Oncology, Department of Internal Medicine, Chungnam National University Hospital, Daejeon, Republic of Korea; 11 Department of Radiation Oncology, Chungnam National University Hospital, Daejeon, Republic of Korea

**Keywords:** Cancer, Computational biology and bioinformatics

## Abstract

This study focused on a novel strategy that combines deep learning and radiomics to predict epidermal growth factor receptor (EGFR) mutations in patients with non-small cell lung cancer (NSCLC) using computed tomography (CT). A total of 1280 patients with NSCLC who underwent contrast-enhanced CT scans and EGFR mutation testing before treatment were selected for the final study. Regions of interest were segmented from the CT images to extract radiomics features and obtain tumor images. These tumor images were input into a convolutional neural network model to extract 512 image features, which were combined with radiographic features and clinical data to predict the EGFR mutation. The generalization performance of the model was evaluated using external institutional data. The internal and external datasets contained 324 and 130 EGFR mutants, respectively. Sex, height, weight, smoking history, and clinical stage were significantly different between the EGFR-mutant patient groups. The EGFR mutations were predicted by combining the radiomics and clinical features, and an external validation dataset yielded an area under the curve (AUC) value of 0.7038. The model utilized 1280 tumor images, radiomics features, and clinical characteristics as input data and exhibited an AUC of approximately 0.81 and 0.78 during the primary cohort and external validation, respectively. These results indicate the feasibility of integrating radiomics analysis with deep learning for predicting EGFR mutations. CT-image-based genetic testing is a simple EGFR mutation prediction method, which can improve the prognosis of NSCLC patients and help establish personalized treatment strategies.

## Introduction

Lung cancer is classified as non-small cell lung cancer (NSCLC) and small cell lung cancer (SCLC) based on the size and shape of the tumor, and NSCLC accounts for approximately 81% of all lung cancers^1^. Currently, various effective targeted therapies have been developed for NSCLC. However, such targeted therapies are more effective for patients with detectable gene mutations; thus, gene mutation tests are essential for patients with NSCLC. Epidermal growth factor receptor (EGFR) mutations are typically observed in patients with NSCLC and are found in approximately 50% of adenocarcinomas^2,3^.

EGFR is a signal transduction protein that regulates the growth and division of cells. When the DNA sequence inside the gene is mutated, the signal transduction pathway can function abnormally, resulting in a rapid proliferation of cancer cells and tumor formation^4^. Therefore, early detection of EGFR mutations is crucial to improve the prognosis of NSCLC patients and select the right treatment to extend their life expectancy^5^.

Typically, in EGFR mutation tests, the peptide nucleic acid (PNA)-mediated polymerase chain reaction (PCR) clamping method, which requires tumor tissue samples, is used^6^. However, through these gene mutation tests, we can only observe a portion of the tumor, and therefore the overall heterogeneity of the tumor cannot be determined. Moreover, a biopsy may increase the risk of metastasis^7^, and limitations, such as technical difficulties and high costs, further impede the applicability of PNA-mediated PCR clamping EGFR tests. Therefore, a noninvasive and simple test method should be developed to overcome the limitations of existing methods^8^.

Medical imaging has emerged as a powerful tool because of its ability to analyze the overall shape and heterogeneity of a carcinoma. Medical imaging techniques facilitate real-time and noninvasive image acquisition throughout the treatment process. In addition, the acquired images contain both basic anatomical and physiological information, as well as precise genetic information^9^. In this study, we developed a novel deep learning and radiomics integrated method to predict the EGFR mutations in NSCLC patients. Radiomics features were extracted from pretreatment contrast-enhanced computed tomography (CT) images of NSCLC patients, and subsequently, these extracted features and tumor images were employed to construct a predictive model.

## Results

The clinical characteristics of the internal and external datasets, which contained 324 and 130 EGFR mutants, respectively, used in this study are summarized in Table 1. In both the internal and external datasets, the sex, height, weight, smoking history, and clinical stage of the patients in the EGFR mutant group were significantly different from those of the patients in the EGFR wild-type group.

**Table 1:** Clinical characteristics.

Clinical characteristics	Internal dataset (n = 847)			External validation dataset (n = 433)		
	EGFR wild type (n = 523)	EGFR mutant (n = 324)	*p*	EGFR wild type (n = 303)	EGFR mutant (n = 130)	*p*
Sex			0.00			0.00
Male	399	213		242	46	
Female	124	111		61	84	
Age (mean)	71.41	69.08	0.00	72.08	69.75	0.03
Height (mean)	163.39	158.66	0.00	163.34	158.04	0.00
Weight (mean)	60.94	58.89	0.00	62.18	59.91	0.07
Smokers	268	68	0.00	190	30	0.00
Family history	13	16	0.09	71	30	1.00
Stage			0.00			0.00
1	185	157		83	53	
2	76	30		39	11	
3	114	28		81	15	
4	148	109		100	52	

The prediction results of the classification models obtained using various input data were compared to derive an optimal model that can classify the EGFR mutant and EGFR wild-type patient groups. The model was trained using a fivefold cross-validation, and the internal validation results were obtained using a hold-out internal test set consisting of Ajou University Medical Center (AJMC) and Chungnam National University Hospital (CNUH) data. External validation results were obtained using the Inha University Hospital (INHA) dataset. The final prediction is calculated by averaging the predictions from each fold's model.

The EGFR mutations were predicted by combining the extracted radiomics and clinical features, and the AUC values on the internal and external validation datasets were approximately 0.73 and 0.70, respectively, as shown in Table 2. The highest predicted results were yielded by the model trained using the acquired tumor images, radiomics features, and clinical data. The prediction results of all models are listed in Table 3, which indicates that Multimodality EfficientNet b7 achieved AUC values of approximately 0.81 and 0.78 in internal and external verifications, respectively. This external verification AUC of 0.78, which is even higher than that of the model combining radiomics and clinical features (AUC = 0.73) (Fig. 4), indicates that the Multimodality EfficientNet b7 model exhibited the highest predictive power. The average and standard deviation of each fold model performance are in Table 4.

**Table 2:** Radiomics-clinical model results.

	Internal validation	External validation
Accuracy	0.6824	0.6443
Precision	0.6061	0.4423
Recall	0.5882	0.7077
F1 score	0.6674	0.6264
AUC	0.7370	0.7038

**Table 3:** Multimodality model results.

	Multimodality EfficientNet b7		Multimodality ResNet34		Multimodality DenseNet 264	
	Internal validation	External validation	Internal validation	External validation	Internal validation	External validation
Accuracy	0.7941	0.7644	0.7588	0.7413	0.7765	0.7067
Precision	0.7018	0.5986	0.6393	0.5592	0.6562	0.5080
Recall	0.6897	0.6538	0.6724	0.6538	0.7241	0.7808
F1 score	0.7700	0.7266	0.7350	0.7055	0.7571	0.6840
AUC	0.8059	0.7760	0.7909	0.7679	0.7934	0.7802

**Table 4:** Mean and standard deviation of 5-fold cross-validation results.

	Multimodality EfficientNet b7		Multimodality ResNet34		Multimodality DenseNet 264	
	Internal validation	External validation	Internal validation	External validation	Internal validation	External validation
Accuracy	0.7153 ± 0.0188	0.7035 ± 0.0283	0.7306 ± 0.0136	0.7109 ± 0.0125	0.7059 ± 0.0268	0.6915 ± 0.0247
Precision	0.6488 ± 0.0224	0.5071 ± 0.0356	0.6886 ± 0.0172	0.5170 ± 0.0173	0.6216 ± 0.0669	0.4922 ± 0.0269
Recall	0.3586 ± 0.0934	0.7062 ± 0.0225	0.3897 ± 0.0876	0.6185 ± 0.0870	0.3414 ± 0.0854	0.7292 ± 0.0171
F1 score	0.6308 ± 0.0441	0.6784 ± 0.0246	0.6531 ± 0.0402	0.6716 ± 0.0157	0.6184 ± 0.0498	0.6703 ± 0.0220
AUC	0.7977 ± 0.0140	0.7619 ± 0.0168	0.7762 ± 0.0191	0.7446 ± 0.0268	0.7861 ± 0.0097	0.7602 ± 0.0177

These are the average and standard deviation of the performance of each fold model.

## Discussion

In this study, EGFR mutations were predicted using a deep learning and radiomics combined technique in which contrast-enhanced CT images and clinical data of NSCLC patients obtained before treatment were provided as input to the deep learning model. In addition, the prediction results of different models were compared to identify the model with the highest prediction and generalization performance. The performance of the predictive model was evaluated using 433 validation set data points and 170 test data points obtained from 1280 NSCLC patients. This study implemented data augmentation, transfer learning, and cross-validation to account for differences in data characteristics between hospitals. The predictive model was trained on the training data through fivefold cross-validation, and the performance of the predictive model was evaluated using 433 hold-out internal test set data points and 170 external test set data points that were not used for training. The predictive model based on combining radiomics features, clinical data, and tumor images obtained AUC values of approximately 0.81 and 0.78 in internal and external validation, respectively. By contrast, the model based on clinical and radiomics features obtained AUC values of 0.73 and 0.70 in internal and external validation, respectively. Therefore, the model that combined clinical data, radiomics features extracted from the CT images, and tumor images achieved the best EGFR mutation prediction performance. These results suggest that a hybrid radiomics model combining deep learning with clinical and radiomics features is a simple and noninvasive tool for classifying EGFR mutations. Existing EGFR mutation tests are expensive and time-consuming, and the reliability and reproducibility of the test results may vary depending on the experimental instrument and conditions. Mutation profiling after biopsy or surgical resection has become a standard and informative medical procedure. However, the potential for molecular testing is significantly limited due to issues such as repetitive tumor sampling, challenging tissue accessibility, difficulties in determining mutational status due to poor DNA quality, and similar factors^10–12^.

CT-image-based genetic testing is a simple EGFR mutation prediction method providing reproducible results and facilitates the early detection of gene mutations, which can improve the prognosis of NSCLC patients and help establish personalized treatment strategies. Thus, this CT-image-based genetic testing method can be combined with deep learning models and used as a non-knowledge-based clinical decision system (CDSS)^13^. Such a non-knowledge-based CDSS can support real-time and rapid decision-making owing to its ability to learn data features and detect new information or patterns^13^. However, these non-knowledge-based CDSSs cannot directly understand the logic of artificial intelligence and are not widely implemented in clinical practice owing to insufficient data^14^. Therefore, a method for evaluating and verifying the reliability of artificial intelligence models should be developed.

Since 2012, radiomics has been applied in oncology to characterize tumor heterogeneity in medical images^15,16^. Radiomics extracts quantitative features from medical images and analyzes them to support diagnosis and treatments. In particular, a texture analysis of CT images helped distinguish tumor lesions with different histopathological characteristics and predict treatment response and patient survival^17^. According to previous studies, features extracted from CT images of lung cancer patients are associated with gene expression patterns and can be utilized to predict EGFR mutation profiles^18–20^. Handcrafted radiomics extracts features from an area inside a tumor; therefore, it does not consider the surrounding tissues and borders. In addition, radiomics is time-consuming and expensive owing to its dependence on accurate tumor boundary annotations. By contrast, deep learning-based radiomics does not require accurate tumor boundary annotations and automatically learns features from image data. Previous studies have confirmed that EGFR mutations can be predicted by extracting high-level abstract features, in addition to low-level visual features of tumors, such as “shape” and “texture,” using deep learning-based techniques^8,21^. However, deep learning-based radiomics cannot explain the decision-making process of a model, and thus its application in the medical field is limited. Recently, a hybrid radiomics method integrating the two methods has been reported to overcome this limitation^22^. Therefore, the present study focused on developing a predictive model combining deep learning with clinical and radiomics characteristics.

According to previous studies, the accuracy of EGFR mutation prediction can be improved by combining clinical and radiomics features. Liu et al. predicted EGFR mutations using CT and clinical data of 298 lung adenocarcinoma patients^19^. The AUC improved from 0.690 to 0.778 when radiomics was added to the clinical model. Furthermore, Zhao et al. predicted EGFR mutations by combining radiomics features and deep learning. The AUC improved from 0.645 to 0.758 when combining radiomics with a deep learning^23^. However, the authors analyzed only the data of patients with adenocarcinoma, among all the NSCLCs, and further validation is required for other lung cancer types. Zhang et al. predicted EGFR mutations by combining clinical features, such as age, sex, clinical stage, and histological classification, of NSCLC patients with those extracted from CT images^24^. The radiomics and clinical feature-integrated predictive model had better prediction accuracy than the model that used only clinical features. These previously reported studies suggest that the accuracy of EGFR mutation prediction can be drastically increased by combining radiomics characteristics with clinical factors in deep learning models.

However, among the clinical factors used in previous studies, tumor sample collection for histological classification requires biopsy. In addition, the region of interest is manually segmented, which is both cost- and time-intensive, and the evaluation criteria may vary according to the empirical judgment of the medical staff. Handcrafted radiomics relies on precise tumor boundary segmentation, which is expensive and involves complex steps (region segmentation, feature extraction, feature selection, and analysis). In this study, deep learning was integrated with radiomics features extracted from CT images and clinical data. Moreover, a predictive model was developed using tumor images to simplify the prediction process while considering the surrounding tissues and borders. However, the prediction accuracy of hybrid radiomics, combining the radiomics technique and deep learning, is still lower than those of existing deep learning models and handcrafted radiomics. Therefore, optimizing the combined method using various data and developing an efficient network structure are necessary.

However, we used only the data of NSCLC patients in Korea for our analysis, even though the EGFR mutation rate may be affected by race. Thus, verifying this generalization using data from patients from different racial backgrounds is required. Furthermore, among all the gene mutations, only EGFR mutations were considered in this study. Mutations in genes, such as anaplastic lymphoma kinase and ROS proto-oncogene 1, which are found in NSCLC patients, are also associated with cancer development and should be considered in future studies. Moreover, the proposed method relies on deep learning algorithms, which do not facilitate interpretation during decision-making. In the future, incorporating explainable artificial intelligence (XAI) and other interpretable models should be explored to explain the functioning and decision-making processes of the predictive model. These XAI and other interpretable models might enhance the reliability of the decision-making process and provide transparency^25^. In addition, in future research, we plan to use Grad-CAM to determine which regions within the images have influenced the classification decisions of the CNN. This study focused on predicting EGFR mutations. Although the predictive model in this study had limited performance for actual clinical application, it appeared to have higher performance (AUC = 0.805) compared to existing previous studies (AUC = 0.778, 0.758)^19,23^. This suggests that hybrid radiomics, which combines clinical features, radiomics, and deep learning, may provide a better prediction method for predicting EGFR mutations. The precision of the predictive model in this study is low at 0.60. However, in a previous study combining radiomics and deep learning, the precision of external validation was 0.53, so the precision was further improved in this study^20^. Considering the inherent characteristics of deep learning, the performance of the predictive model might be improved by increasing the dataset size. Precision is affected by sample size, so further studies within larger populations are needed. This would enable the model to learn and generalize a broader spectrum of patterns and features^26^. In future work, we plan to learn and generalize broader patterns and features through public datasets and various pre-trained models.

In conclusion, this study developed a simple and noninvasive method that combines tumor images, radiomics features, and clinical data extracted from pretreatment CT scans of NSCLC patients using deep learning models to predict EGFR mutations. EGFR mutation increases the risk of cancer recurrence in patients with NSCLC and can also be used as an indicator to determine treatment strategies. Therefore, further studies should be conducted to improve the accuracy of the proposed model and address the data construction disadvantages.

## Methods

### Dataset

**Figure 1:**
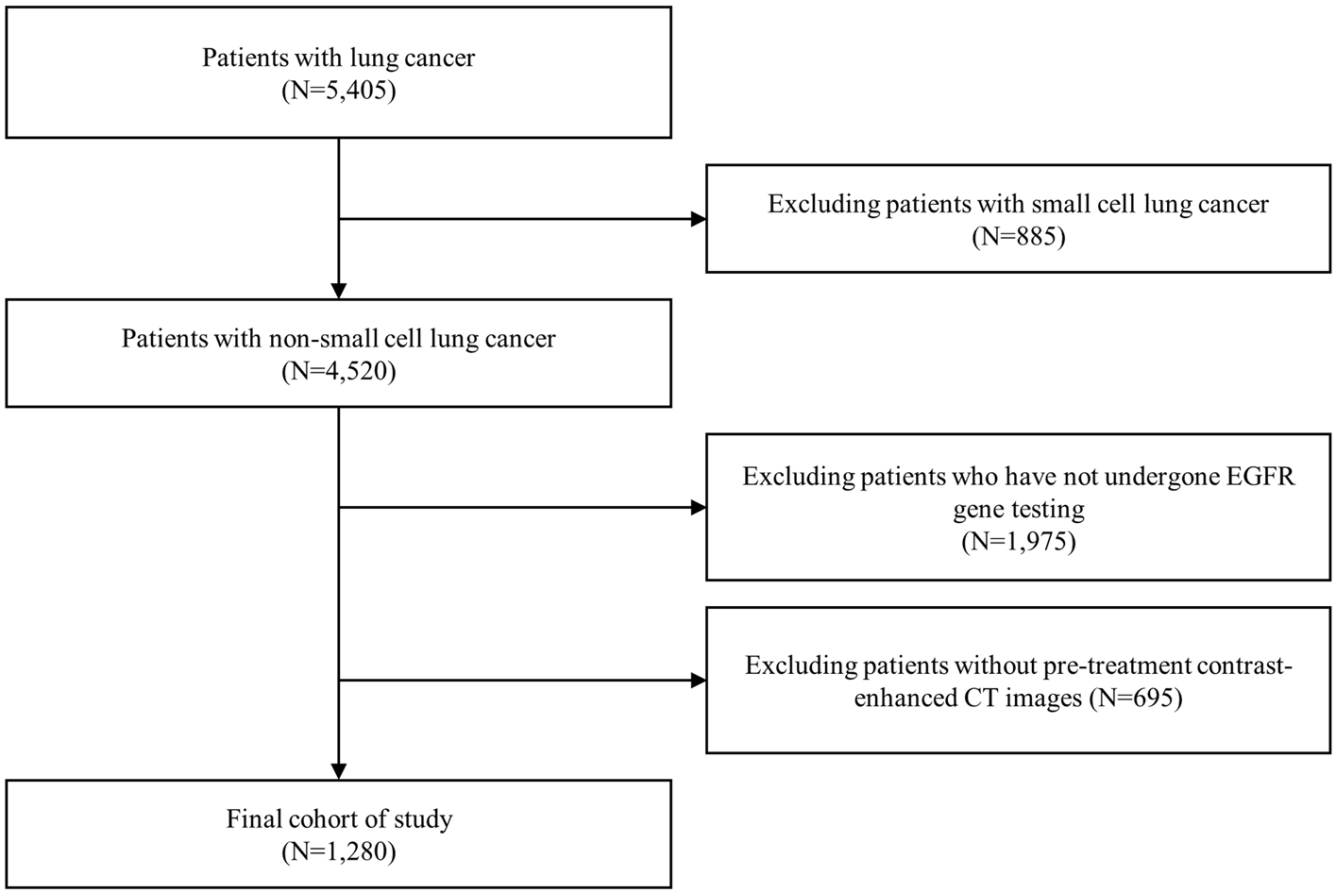
Diagram of the patient selection process.

CT images and clinical data of 5405 patients with lung cancer were retrospectively collected from three domestic hospitals in South Korea, namely the Ajou University Hospital, Inha University Hospital, and Chungnam National University Hospital. The dataset was externally validated by the Telecommunications Technology Association of Korea. Figure 1 includes a flowchart describing patient selection. Among these patients, 1280 individuals with NSCLC, who underwent EGFR mutation testing and had pretreatment CT scans, were selected for the final analysis. Among these patients, 1280 individuals with NSCLC, who underwent EGFR mutation testing and had pretreatment CT scans, were selected for the final analysis. Clinical data included sex, age, family history, clinical stage, height, weight, and tumor location. The collected data consisted of 454 cases of EGFR mutations and 826 cases of wild-type EGFR without mutations. At each of the participating hospitals, namely, Ajou University Hospital, Chungnam National University Hospital, and Inha University Hospital, Institutional Review Board (IRB) approval was obtained, and the approval numbers are as follows: Ajou University Hospital (AJOUIRB-DB-2023-196), Chungnam National University Hospital (CNUH-IRB-2022-10-026), and Inha University Hospital (INHA-IRB-2022-08-024-000). Furthermore, the requirement for informed consent from all participants was waived by the IRB at Ajou University Hospital, Chungnam National University Hospital, and Inha University Hospital because of the retrospective nature of this study. All methods were performed in accordance with the Declaration of Helsinki.

**Figure 2:**
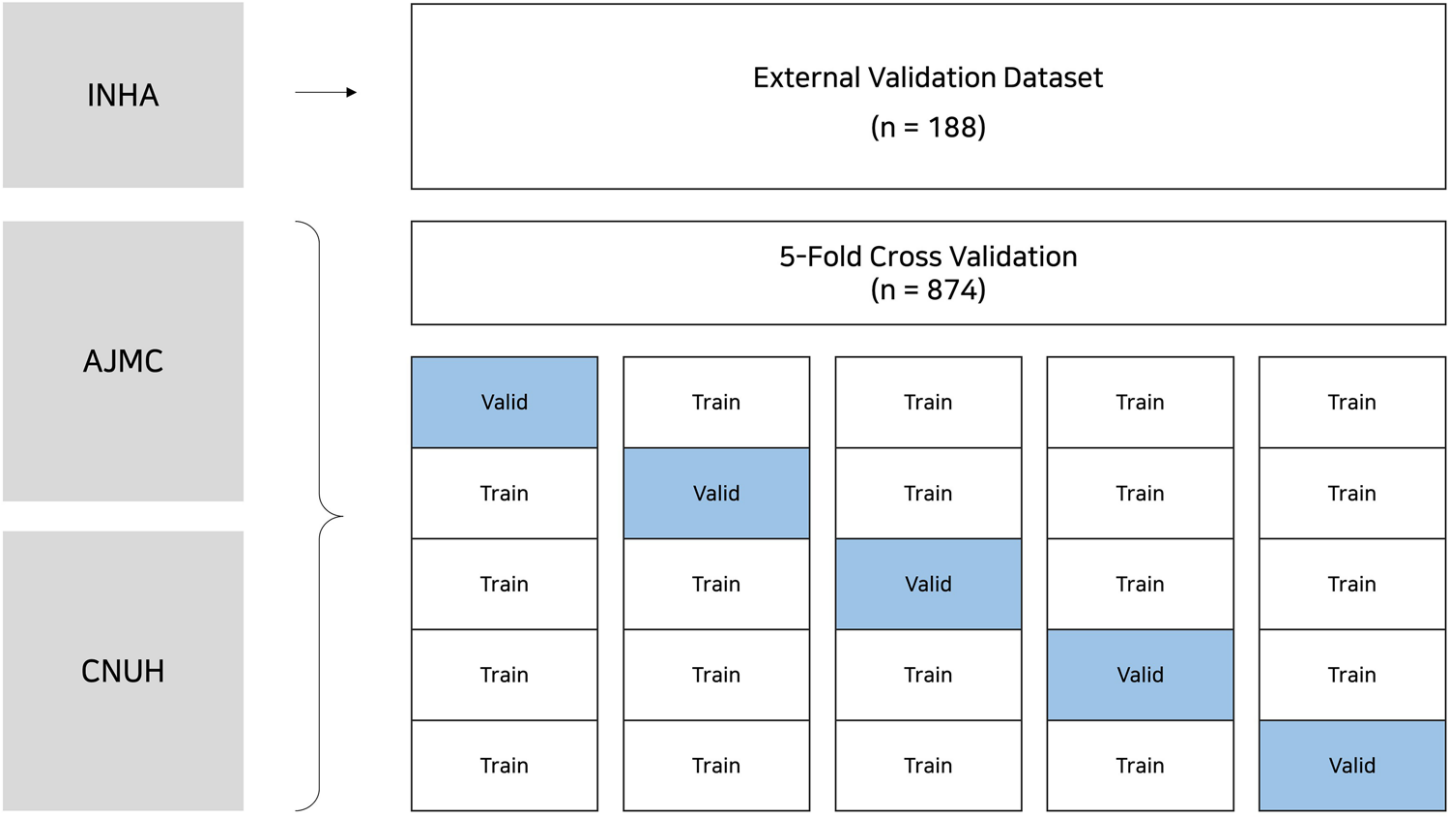
Validation and test structure of the NSCLC dataset for deep learning. INHA, Inha University Hospital; AJMC, Ajou University Medical Center; CNUH, Chungnam National University Hospital.

To preprocess the clinical data, first, categorical variables, such as gender, family history, and smoking history, were converted into binary variables via one-hot encoding. In the case of sex, male and female were coded as 1 and 0, respectively, while family history and no family history were coded as 1 and 0, respectively. Furthermore, missing values were replaced with the median of the variable and normalized by min–max scaling. Subsequently, to preprocess the CT images (which had a depth of 16 bits, and the pixel value was normalized between 0 and 1 for the generalization performance of the model), modality and value-of-interest (VOI) lookup tables (LUTs) and histogram flattening were applied. The modality and VOI LUTs were employed to determine the changes in brightness, contrast, and size data obtained using different CT imaging equipment and protocols, and histogram flattening enabled contrast enhancement and clarity. Furthermore, the data obtained from the Chungnam National University Hospital and Ajou University Hospital data were used for training and the internal validation of the proposed model, respectively, whereas those obtained from the Inha University Hospital were used as the external validation dataset (Fig. 2).

### Radiomics feature extraction

To segment the tumor regions visible in the original CT images, a fully automatic segmentation technique was implemented using a universal network (i.e., U-Net) model pretrained using the Lung-PET-CT-Dx dataset, which was obtained from the Cancer Imaging Archive^27^. The automatically segmented lung tumor masks were saved as binary images and used in the analysis. In this study, we used the PyRadiomics module of Python to extract the first-order statistics and secondary statistical features, namely shape (3D), shape (2D), gray level co-occurrence matrix, gray level size zone matrix, gray level run length matrix, neighboring gray-tone difference matrix, and gray level dependence matrix. In total, 107 radiomics features were extracted and normalized by min–max scaling.

### Development of deep learning model

To obtain the CT image, a 224 × 224 tumor area was cut and converted into a 112 × 112 area. Subsequently, 64 images of the central tumor were captured. These tumor images were input into convolutional neural network (CNN) models, i.e., EfficientNet b7, ResNet 34, and DenseNet264. EfficientNet uniformly scales all dimensions of a network, including depth, width, and resolution, to improve accuracy and efficiency^28^. In this study, the largest model in the EfficientNet series, EfficientNet b7, was employed. ResNet34 is a model that addresses the problems of gradient vanishing and exploding by introducing the Residual Network (ResNet) technique^29^. Lastly, DenseNet incorporates the concept of dense connectivity, enabling each layer within the network to be directly connected to the output of the previous layer^30^. The model was trained through transfer learning, utilizing pre-trained model weights based on the ImageNet dataset^31^. The hyperparameters were set as follows: the optimizer employed was AdamW (Adam with Weight Decay), with a learning rate of 0.00001, and the scheduler used was CosineAnnealingLR (Cosine Annealing Learning Rate Scheduler). The CNN models were used to extract the image features. The model with the highest performance was selected to construct the predictive model. Radiomics features and clinical characteristics were converted into 512 dimensions through linear transformation, and then BatchNorm (Batch Normalization) and Dropout were applied.

**Figure 3:**
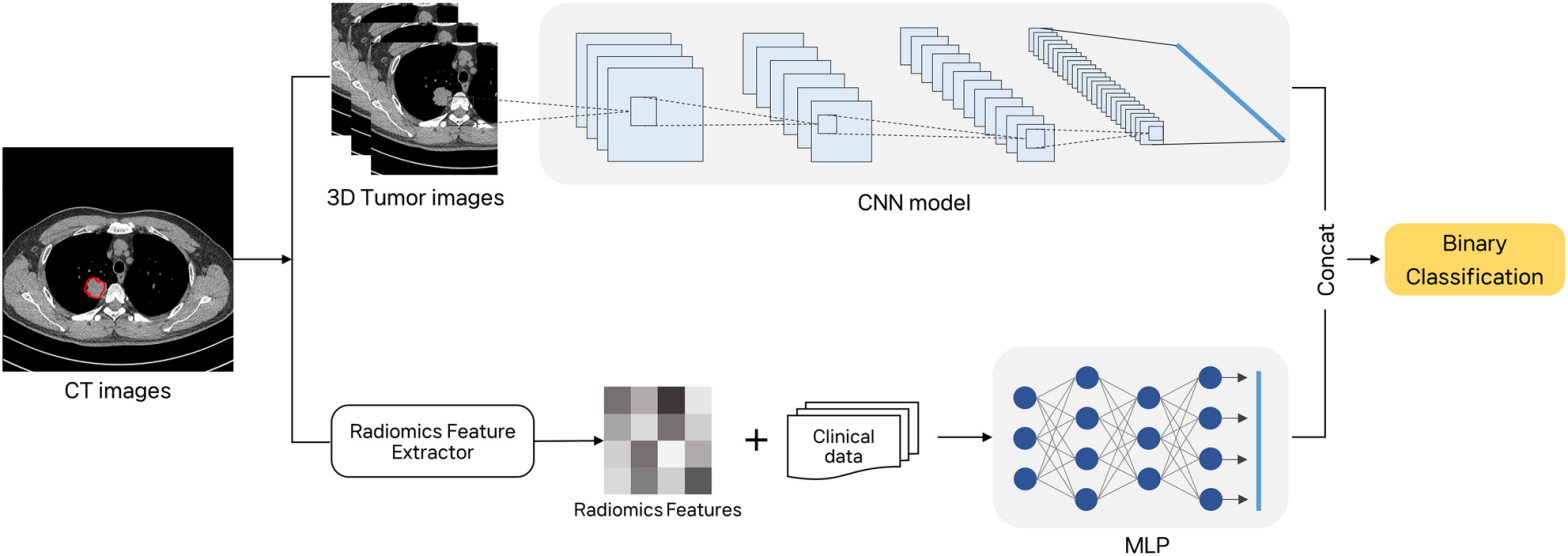
Overview of the deep learning architecture.

The tumor image, provided as input to the CNN model, was 3D augmented via image rotation, change in scale position, noise addition (random noise), and blurring to prevent overfitting by the model and improve its generalization performance. The image features were extracted by combining the linearly transformed radiomics and clinical features using a multilayer perceptron. A 1024-dimensional vector was input into the model, while a linear layer and a layer subjected to the hyperbolic tangent (tanh) function were used as the hidden layers. After passing through the hidden layer, the final predicted output was processed by the linear layer to classify the EGFR mutant and EGFR wild-type cancers (Fig. 3). Furthermore, to compare the results based on input data, a deep learning model was also constructed using only radiomics and clinical features.

### Statistical analyses

**Figure 4:**
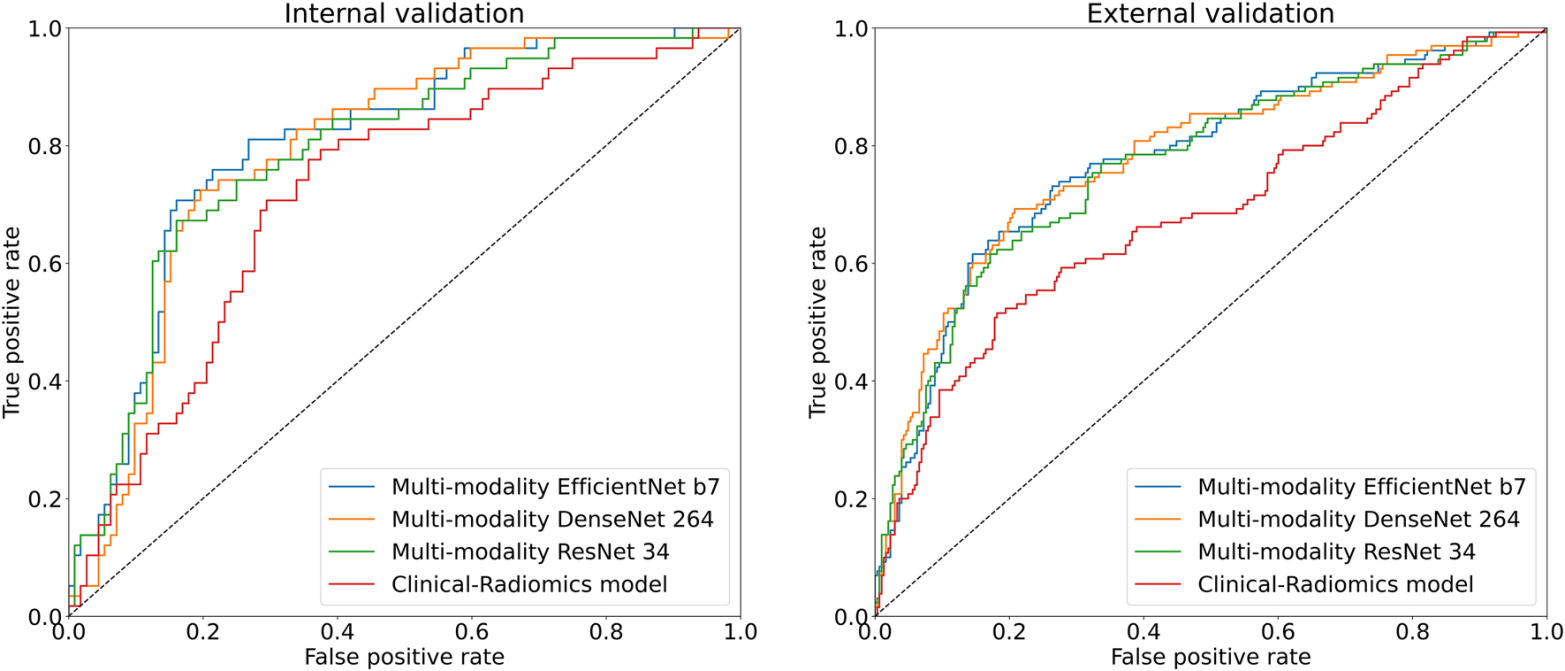
ROC curves for the (**A**) internal and (**B**) external validation datasets.

We evaluated the performance of the classification models using objective evaluation metrics, including specificity, precision, sensitivity, F1-score, and accuracy, whose mathematical foundations are based on the true positive (TP), true negative, false negative, and false positive (FP) values of the models’ predictions. In addition, we used the “area under the curve” (AUC) of the receiver operating characteristic (ROC) curve to evaluate the binary-classification performance of the deep learning algorithm. To plot the ROC curve, we calculated the TP rate (sensitivity) and FP rate (1-specificity) with different predicted probability thresholds and then determined the AUC values (Fig. 4).

All the statistical analyses were performed on Ubuntu 18.04 with Pandas (version 1.5.3), Scikit-learn (version 1.2.1), NumPy (version 1.23.5), Matplotlib (version 3.6.3), and PyTorch (version 1.13.1) using the OpenCV-python (version 4.7.0.68) package. The model structures were developed on a graphics processing unit server (450.51.05) with NVIDIA Tesla V100 (32 GB * 4) and Xeon Gold 6248 (Intel).

### Ethical statement

The Institutional Review Board of Ajou University Hospital approved this study (IRB No. AJOUIRB-DB-2023-196). Further, informed consent from all participants was waived by the IRB because of the retrospective nature of this study.

## Data Availability

The datasets generated and/or analyzed in this study are available from the corresponding author upon reasonable request.

## Code availability

To train the classification model in this study, we used the publicly available pytorch training script available at https://github.com/VemundFredriksen/LungTumorMask.
